# Alogliptin ameliorates postprandial lipemia and postprandial endothelial dysfunction in non- diabetic subjects: a preliminary report

**DOI:** 10.1186/1475-2840-12-8

**Published:** 2013-01-09

**Authors:** Yoko Noda, Toru Miyoshi, Hiroki Oe, Yuko Ohno, Kazufumi Nakamura, Norihisa Toh, Kunihisa Kohno, Hiroshi Morita, Kengo Kusano, Hiroshi Ito

**Affiliations:** 1 Department of Cardiovascular Medicine, Okayama University Graduate School of Medicine, Dentistry and Pharmaceutical Sciences, Okayama, Japan; 2 Department of Cardiovascular Therapeutics, Okayama University Graduate School of Medicine, Dentistry and Pharmaceutical Sciences, 2-5-1, Shikata-cho, Okayama, 700-8558, Japan; 3 Center of Ultrasonic Diagnostics, Okayama University Hospital, Okayama, Japan

**Keywords:** Dipeptidyl peptidase IV inhibitor, Postprandial lipid, Triglyceride-rich lipoprotein, Endothelial dysfunction, Alogliptin

## Abstract

**Background:**

Postprandial hyperlipidemia impairs endothelial function and participates in the development of atherosclerosis. We investigated the postprandial effects of a dipeptidyl peptidase IV inhibitor, alogliptin, on endothelial dysfunction and the lipid profile.

**Methods:**

A randomized cross-over trial design in 10 healthy volunteers (8 males and 2 females, 35 ± 10 years) was performed. The postprandial effects before and after a 1-week treatment of 25 mg/day alogliptin on endothelial function were assessed with brachial artery flow-mediated dilation (FMD) and changing levels of lipids, apolipoprotein B48 (apoB-48), glucose, glucagon, insulin, and glucagon-like peptide-1 (GLP-1) during fasting and at 2, 4, 6, and 8 h after a standard meal loading test.

**Results:**

Alogliptin treatment significantly suppressed the postprandial elevation in serum triglyceride (incremental area under the curve [AUC]; 279 ± 31 vs. 182 ± 32 mg h/dl, p = 0.01), apoB-48 (incremental AUC; 15.4 ± 1.7 vs. 11.7 ± 1.1 μg h/ml, p = 0.04), and remnant lipoprotein cholesterol (RLP-C) (incremental AUC: 29.3 ± 3.2 vs. 17.6 ± 3.3 mg h/dl, p = 0.01). GLP-1 secretion was significantly increased after alogliptin treatment. Postprandial endothelial dysfunction (maximum decrease in%FMD, from −4.2 ± 0.5% to −2.6 ± 0.4%, p = 0.03) was significantly associated with the maximum change in apoB-48 (r = −0.46, p = 0.03) and RLP-C (r = −0.45, p = 0.04).

**Conclusion:**

Alogliptin significantly improved postprandial endothelial dysfunction and postprandial lipemia, suggesting that alogliptin may be a promising anti-atherogenic agent.

## Introduction

Large prospective studies have shown that non-fasting postprandial triglyceride (TG) concentrations predict cardiovascular risk better than fasting TG concentrations and that this relationship is independent of traditional coronary risk factors [1,2]. TG-rich lipoproteins, which consist of chylomicrons assembled by TG, dietary cholesterol, and apolipoprotein B-48 (apoB-48), are highly atherogenic and contribute to the development of coronary heart disease. Thus, the increased risk of cardiovascular events associated with non-fasting TG concentrations may reflect atherogenic properties of TG-rich lipoproteins generated during the postprandial period [3]. Studies have shown that postprandial lipemia contributes to the production of proinflammatory cytokines and oxidative stress, resulting in endothelial dysfunction even in healthy normolipidemic people [4,5]. Furthermore, other studies demonstrated that postprandial hyperlipemia caused by oral fat intake impairs endothelial dysfunction as detected with flow-mediated dilatation (FMD) of the brachial artery in healthy volunteers. This endothelial dysfunction is associated with postprandial TG-rich lipoproteins [6,7]. Therefore, identification of novel therapeutic approaches that would beneficially affect postprandial concentrations of lipids is of great interest.

Alogliptin is a potent and selective inhibitor of dipeptidyl peptidase IV (DPP-4) and has been shown to reduce fasting and postprandial glucose levels in patients with type 2 diabetes, presumably by inhibiting the inactivation of glucagon-like peptide-1 (GLP-1) and glucose-dependent insulinotropic polypeptide (GIP), thereby improving islet function [8-10]. Recent clinical studies have reported that DPP-4 inhibitors such as vildagliptin and sitagliptin improve postprandial atherogenic TG-rich lipoprotein levels in patients with type 2 diabetes [11,12]. However, the effects of other DPP-4 inhibitors on postprandial lipemia-induced endothelial dysfunction have not been fully evaluated.

The aim of this study was to investigate the effects of alogliptin on postprandial triglyceride (TG)-rich lipoprotein and postprandial lipemia-induced endothelial dysfunction.

## Methods

### Participants

Ten volunteers, including eight men and two women, were recruited. The study consisted of two 1-week cross-over treatment periods with 25 mg/day alogliptin and placebo in random order, including a 1-week washout period between the two phases. All participants underwent medical check-ups. None of the 10 volunteers had hypertension, impaired glucose tolerance, dyslipidemia, or cerebrovascular or cardiovascular disease, but three volunteers were current smokers. Family histories were obtained from medical interviews. Impaired glucose tolerance was defined as 2-h glucose level of 140–199 mg/dl after the meal loading test [13], and dyslipidemia was defined as one or more of the following criteria at the fasting state: (1) serum triglyceride ≥150 mg/dL, (2) HDL-cholesterol <40 mg/dL, and (3) LDL-cholesterol ≥140 mg/dL [14]. Lipid profiles and endothelial function, which was assessed with brachial artery FMD during fasting and at 2, 4, 6, and 8 h after an oral cookie loading test, were determined following each phase of treatment. Participants were instructed to take one tablet after their morning meal. This study was approved by the Ethics Committee of Okayama University Graduate School of Medicine, Dentistry, and Pharmaceutical Sciences, and written informed consent was obtained from all volunteers before beginning the protocol.

### Study protocol

After overnight fasting for at least 8 h, a cookie test was performed. The cookie consisted of 75 g carbohydrate (flour starch and maltose), 28.5 g fat (butter), and 8 g protein for a total of 592 kcal per a carton (SARAYA Corp., Osaka, Japan) [15]. Participants were instructed to ingest the cookie with water within 20 min. Time measurement was started when half the cookie had been ingested. Venous blood samples were drawn, and endothelium-dependent vascular function, as assessed with FMD of the brachial artery, was determined during the fasting state before cookie ingestion and at 2, 4, 6, and 8 h after the cookie load. Endothelium-independent dilation, as assessed with nitroglycerin-mediated dilation (NMD), was also measured during fasting before cookie ingestion and 8 h after the cookie load. For the 8 h after eating the cookie, the participants were instructed not to eat anything else. Measurements of FMD and NMD were performed by the same technician, who was blinded to the study design and medication status.

### Measurement of biochemical parameters

The following parameters during fasting before cookie ingestion were measured: serum total cholesterol (Total-C), TG, low-density lipoprotein cholesterol (LDL-C), high-density lipoprotein cholesterol (HDL-C), remnant lipoprotein cholesterol (RLP-C), apoB-48, adiponectin, soluble vascular cell adhesion molecule 1 (VCAM-1), and plasma glucose levels. HbA1c levels were measured using high-performance liquid chromatography. Concentrations of fasting plasma insulin were measured using a chemiluminescent enzyme immunoassay. Lipid profiles and other markers were measured at SRL Co., Ltd., Tokyo, Japan. Homeostasis model assessment of insulin resistance (HOMA-IR) was calculated as [fasting plasma glucose (mg/dl) × fasting plasma insulin (μIU/ml)/405]. Serum Total-C, TG, LDL-C, HDL-C, RLP-C, apoB-48, plasma glucose, and soluble VCAM-1 were measured at 2, 4, 6, and 8 h after the cookie load. To compare the postprandial changes in these parameters before and after treatment for 4 weeks, the area under the curve (AUC) was calculated using the trapezoidal method.

### FMD measurement

Endothelium-dependent and -independent dilation was assessed as a parameter of vasodilation according to the guidelines for ultrasound assessment of FMD of the brachial artery [16]. Using a 10-MHz linear-array transducer probe (Unex Company Ltd., Nagoya, Japan), longitudinal images of the brachial artery at baseline were recorded with a stereotactic arm, and measurements of artery diameter were made after supine rest for ≥5 min. The diameter of the artery was measured from clear anterior (media-adventitia) and posterior (intima-media) interfaces, which were manually determined. Then, suprasystolic compression (50 mmHg higher than systolic blood pressure) was performed at the right forearm for 5 min, and measurements of artery diameter were made continuously from 30 s before to ≥2 min after cuff release. After ≥10 min of rest from FMD measurement, artery diameter at baseline and for 5 min after administration of 0.3 mg sublingual nitroglycerin was also measured. Maximum vasodilation was then evaluated from the change in artery diameter after release of occlusion (%FMD) and after administration of nitroglycerin (%NMD).

### Statistical analysis

Sample size was determined based on the estimated FMD reported in another recent study [7]. We assumed that the mean improvement in postprandial%FMD was 2.7% and the standard deviation (SD) was 2.0%. To use a two-sided test for differences, a minimal sample size of 10 participants was required in each group to detect statistical differences in%FMD with a power of 80% and an α-type error of 5% in statistical analysis. Results and data in the figures are expressed as the mean ± standard error (SE). Categorical variables were compared using the χ^2^ test or Fisher’s exact test. Differences in lipid profile and endothelial function between the two groups were compared using the Wilcoxon signed-ranks test. Pearson correlation coefficients were used to assess the relationships between maximum reduction in postprandial%FMD and lipid profiles. Values of p < 0.05 were considered significant.

## Results

### Characteristics of participants

The mean age and body mass index of these volunteers were 35 ± 10 years and 23.9 ± 4.1 kg/m^2^, respectively. Participants maintained their weight throughout the study. Table 1 shows the lipid/lipoprotein profile and the glycemic parameters of the participants following each 1-week phase of either control or treatment with 25 mg/day alogliptin. During fasting, 1-week treatment with alogliptin did not affect the lipid/lipoprotein profile or the levels of adiponectin and soluble VCAM-1. Alogliptin significantly increased GLP-1 levels with no significant impact on fasting blood glucose or HOMA-IR (insulin resistance). No significant differences were observed in systolic and diastolic blood pressure following alogliptin treatment.

**Table 1:** Characteristics of participants

	**Control (n = 10)**	**Alogliptin (n = 10)**	**p value**
Age (years)	35 ± 10	-	-
Male (%)	8 (80)	-	-
Current Smoker (%)	3(30)	-	-
HbA1c (%)	4.6 ± 0.3	-	-
BMI (kg/m^2^)	23.9 ± 4.1	23.9 ± 3.4	0.86
Systolic blood pressure (mmHg)	122 ± 3	121 ± 3	0.35
Diastolic blood pressure (mmHg)	71 ± 2	71 ± 2	0.99
Heart rate (beats/min)	62 ± 3	61 ± 2	0.20
Total-C (mg/dl)	185.3 ± 10.6	180.7 ± 9.9	0.58
LDL-C (mg/dl)	103.2 ± 8.9	102.2 ± 9.3	0.72
HDL-C (mg/dl)	66.3 ± 3.1	64.2 ± 3.4	0.29
TG (mg/dl)	73.7 ± 10.2	63.4 ± 7.9	0.08
RLP-C (mg/dl)	8.4 ± 1.2	6.7 ± 0.8	0.28
ApoB-48 (μg/ml)	2.5 ± 0.3	2.2 ± 0.2	0.19
Glucose (mg/dl)	93.0 ± 2.0	94.3 ± 2.1	0.54
Glucagon (pg/ml)	63.5 ± 5.4	59.4 ± 2.8	0.44
Insulin (μIU/ml)	4.8 ± 0.8	4.9 ± 1.3	0.38
HOMA-IR	1.1 ± 0.2	1.1 ± 0.3	0.51
GLP-1 (pmol/l)	3.2 ± 0.2	5.2 ± 0.7	0.03
Adiponectin (μg/ml)	9.6 ± 0.8	9.7 ± 0.7	0.31
Soluble VCAM-1 (ng/ml)	629 ± 72	606 ± 50	0.76

Data are the mean ± SE or frequency counts (percentages), as appropriate. BMI, body mass index; LDL-C, low-density lipoprotein cholesterol; HDL-C, high-density lipoprotein cholesterol; TG, triglyceride; RLP-C, remnant lipoprotein cholesterol; ApoB-48, apolipoprotein B-48; HOMA-IR, homeostasis model assessment of insulin resistance; GLP-1, glucagon-like peptide-1; VCAM-1, vascular cell adhesion molecule-1.

### Postprandial lipid and glucose homeostasis

**Figure 1:**
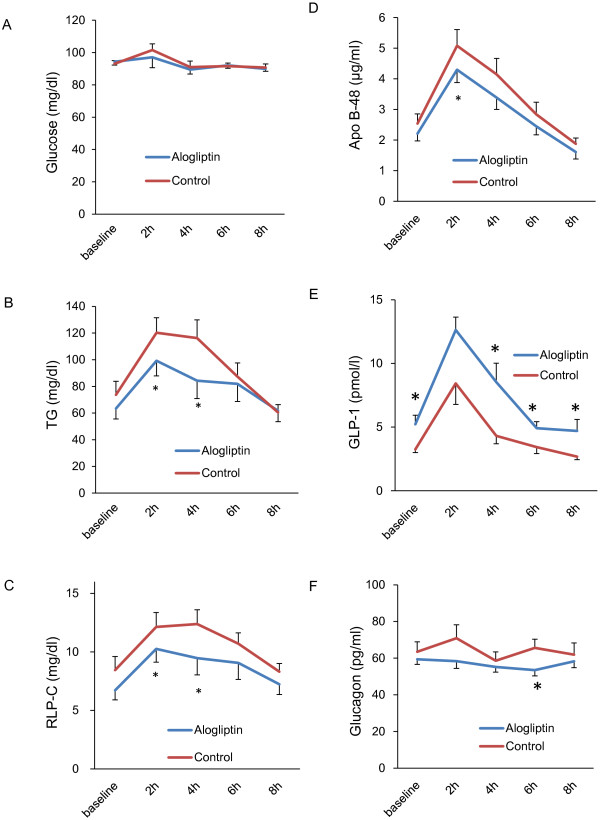
**The serial change in glucose (A), triglyceride (TG) (B), remnant lipoprotein cholesterol (RLP-C) (C), apolipoprotein B-48 (ApoB-48) (D), glucagon-like peptide-1 (GLP-1) (E), and glucagon (F).** *p<0.05 vs. control group.

The levels of lipid/lipoprotein, parameters of glucose homeostasis, and soluble VCAM-1 in the postprandial state are shown in Figure 1 and Additional file 1: Table S1. The serial changes in parameters following each 1-week phase of either control or treatment with alogliptin are shown in Figure 1. In the control group, the postprandial levels of serum TG, RLP-C, apoB-48, and GLP-1 increased and peaked at 2 or 4 h and then returned to baseline at 8 h. The levels of glucose, total-C, LDL-C, HDL-C, and soluble VCAM-1 did not change significantly during the postprandial state. Therefore, the nominal maximum changes and the incremental AUCs of TG, RLP-C, ApoB-48, glucose, and GLP-1 were calculated. The total AUCs of total-C, LDL-C, HDL-C, glucagon, and soluble VCAM-1 were also calculated for comparison.

The maximum changes in postprandial TG, RLP-C, ApoB-48, and GLP-1 were significantly smaller in the alogliptin group compared with the control group (Table 2). The incremental AUCs of serum TG, RLP-C, and apoB-48 were significantly lower in the alogliptin group than in the control group (incremental AUC of TG: 279 ± 31 mg vs. 182 ± 32 mg h/dl, p = 0.01; RLP-C: 29.3 ± 3.2 vs. 17.6 ± 3.3 mg h/dl, p = 0.01; apoB-48: 15.4 ± 1.7 vs. 11.7 ± 1.1 μg h/ml, p = 0.04). No differences in the total AUCs of total-C, LDL-C, or HDL-C were observed between the alogliptin group and the control groups (total AUC of total-C: 1452 ± 252 vs. 1489 ± 248 mg h/dl, p = 0.68, LDL-C: 814 ± 228 vs. 819 ± 218 mg h/dl, p = 0.72, HDL-C: 514 ± 71 vs. 532 ± 79 mg h/dl, p = 0.10).

**Table 2:** Maximum change in lipids and glucose metabolism in the alogliptin and control groups

	**Control**	**Alogliptin**	**p**
TG (mg/dl)	59.7 ± 7.8	48.3 ± 6.4	0.01
RLP-C (mg/dl)	6.1 ± 0.6	4.8 ± 0.7	0.02
ApoB-48 (μg/ml)	4.0 ± 0.4	2.8 ± 0.2	0.01
Glucose (mg/dl)	20.5 ± 2.4	20.4 ±3.9	0.51
Insulin (μIU/ml)	19.4 ± 6.3	18.2 ± 4.8	0.87
GLP-1 (pmol/l)	5.4 ± 1.3	10.2 ± 1.3	0.04

Data are the mean ± SE. TG, triglyceride; RLP-C, remnant lipoprotein cholesterol; ApoB-48, apolipoprotein B-48; GLP-1, glucagon-like peptide-1.

Regarding parameters of glucose homeostasis, there were no significant differences in the incremental AUCs of glucose or insulin between the alogliptin and control groups (incremental AUC of glucose: 78 ± 15 vs. 77 ± 8 mg h/dl, p = 0.58, insulin: 48.3 ± 10.9 vs. 49.4 ± 14.7, p = 0.96), although the incremental AUC of GLP-1 was increased in the alogliptin group (33.8 ± 5.2 vs. 18.2 ± 4.4 pmol h/l, p = 0.02). The total AUC of glucagon was decreased significantly after alogliptin treatment (451.9 ± 21.9 vs. 515.6 ± 30.4 pg h/ml, p = 0.02). No significant difference was observed in the level of soluble VCAM-1 between the alogliptin and control groups (4931 ± 1528 vs. 4935 ± 1411 ng h /ml, p = 0.79).

### Postprandial endothelial function

**Figure 2:**
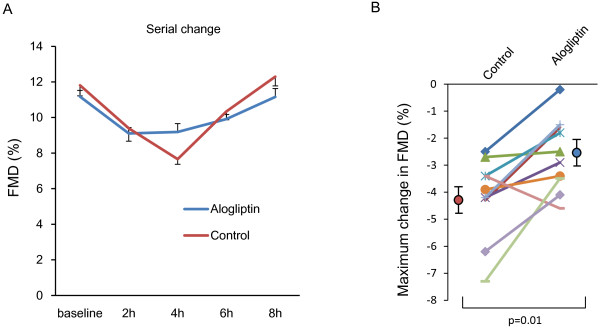
**The serial change in endothelial function after cookie ingestion in the alogliptin and control groups.** FMD, flow-mediated dilation (**A**), and maximum decrease in the alogliptin and control groups (**B**).

Comparison of postprandial endothelial function, which was assessed as%FMD, between the control and alogliptin groups is shown in Figure 2. In the control group, postprandial%FMD decreased significantly, reached the lowest level at 4 h (from 11.8 ± 0.6 to 7.7 ± 0.3%, fasting vs. 4 h, p < 0.01), and recovered at 8 h (from 11.8 ± 0.6 to 12.3 ± 0.5%, fasting vs. 8 h, p = 0.15). The maximum decrease in postprandial%FMD was significantly improved after alogliptin treatment compared to the control group (−2.6% vs. −4.2%, p = 0.03). In the analysis of all data using the control and alogliptin groups, linear regression analysis revealed that the maximum reduction in postprandial%FMD was significantly associated with maximum increases in postprandial TG, RLP-C, and apoB-48 concentrations (TG: r = −0.45, p = 0.04; RLP-C: r = −0.45, p = 0.04; apoB-48: r = −0.47, p = 0.03), and tended to be correlated with the maximum change in GLP-1 (r = 0.39, p = 0.08). However, the maximum reduction in postprandial%FMD was not associated with the maximum change in postprandial LDL-C, HDL-C, glucose, or soluble VCAM-1.

## Discussion

This study demonstrated that alogliptin treatment significantly reduced postprandial levels of intestinally derived apo-B48–containing lipoproteins, which were induced by a conventional oral cookie loading test (28.5 g fat per person), and that alogliptin improved postprandial lipemia-induced endothelial dysfunction. Considering the significant association between the beneficial change in endothelial dysfunction and the decrease in TG-rich lipoproteins, cardiovascular risk, especially associated with postprandial lipemia, may be reduced with long-term treatment with alogliptin.

Our study with the oral cookie test showed that a greater increase in TG-rich lipoprotein, but not glucose, was correlated with postprandial FMD impairment in healthy volunteers. This study did not include people with impaired glucose tolerance and dyslipidemia. Our finding suggests that the impairment in endothelial dysfunction induced by postprandial lipemia is more common than that induced by postprandial hyperglycemia in the general population. As reported in other studies, postprandial hyperglycemia induces endothelial dysfunction, especially in patients with diabetes mellitus or glucose intolerance. In these patients, an increase in glucose is also associated with postprandial endothelial dysfunction [17]. Furthermore, patients with diabetes mellitus often show dyslipidemia including postprandial hyperlipemia. Therefore, our results suggest that alogliptin therapy possibly improves glucose metabolism as well as postprandial hyperlipemia and postprandial endothelial dysfunction in patients with diabetes mellitus.

Previous studies showed that DPP-4 inhibitors such as vildagliptin and sitagliptin decrease postprandial TG, RLP-C, and apoB-48 levels after a fat-loading test in patients with type 2 diabetes [11,12]; however, our study is the first to show that alogliptin reduces the postprandial increase in triglyceride-rich lipoproteins in non-obese nondiabetic subjects.

This study was not designed to examine the molecular mechanisms underlying the effect of alogliptin on postprandial hyperlipemia, but several mechanisms are possible. A study showed that GLP-1 influences intestinal TG absorption [18], potentially by inhibiting gastric lipase [19]. Animal studies have shown that DPP-4 inhibition or GLP-1 receptor agonists significantly reduce intestinal secretion of TG, cholesterol, and apoB-48, suggesting that GLP-1 may directly regulate lipoprotein assembly or the secretion in enterocytes [20]. As shown in our previous study, administration of ezetimibe, an inhibitor of cholesterol absorption, improves postprandial lipemia-induced endothelial dysfunction, mainly due to suppression of postprandial TG-rich lipoproteins [7]. In our current study, the maximum decrease in FMD was significantly associated with the maximum change in TG, RLP-C, and apoB-48, but not glucose. Although further studies are needed to determine the extent to which decreased TG absorption and increased chylomicron clearance contribute to the alogliptin-induced reduction in postprandial lipid response, these findings support our concept that alogliptin markedly decreases the levels of postprandial TG-rich lipoproteins, resulting in prevention of postprandial lipemia-induced endothelial dysfunction.

Regarding another proposed mechanism of improvement in postprandial endothelial dysfunction, an increase in active GLP-1 after alogliptin administration may have direct favorable effects on vascular function. An experimental study reported that sitagliptin improves endothelial function and reduces proinflammatory cytokines and atherosclerosis in apoE-deficient mice [21]. Another experimental study showed that a GLP-1 analog reduces oxidative stress in endothelial cells [22]. A clinical study showed that vildagliptin improves endothelium-dependent vasodilatation as determined by plethysmography in patients with type 2 diabetes in a fasting state [23]. Postprandial inflammation and oxidative stress, which are well known to affect the metabolism of nitric oxide and the release of vasoconstrictive mediators, result in endothelial dysfunction [5,24]. In our study, we did not examine the effect of alogliptin on postprandial oxidative stress. We evaluated the levels of soluble VCAM-1 as a marker of vascular inflammation, but no significant difference was observed. Therefore, we cannot conclude whether the administration of alogliptin improves postprandial inflammation and oxidative stress. A previous study compared the effects of α-glucosidase inhibitors on postprandial glucose/lipid metabolism and endothelial dysfunction in patients with diabetes and showed that miglitol was better than voglibose regarding a greater reduction in triglyceride and a greater induction of GLP-1 [25]. In addition, another study showed that a single dose of exenatide improves postprandial endothelial dysfunction in individuals with impaired glucose tolerance and recent-onset type 2 diabetes. These clinical studies indicate that GLP-1 has direct favorable effects on postprandial endothelial dysfunction [26]. In our current study, no difference was observed between the alogliptin and control groups in glucose levels at 2 h, which was probably due to our use of healthy volunteers. Even though we did not compare alogliptin and other glycemic control agents in this study, a greater spike in GLP-1 after the fat-loading test and/or a greater secretion of GLP-1 after each meal for 1 week in the alogliptin group may have partly contributed to the protective effect on postprandial endothelial dysfunction.

A clinical report showed that sitagliptin treatment for 3 months increases adiponectin levels in patients with diabetes mellitus [27]. This report also suggests that improvement of endothelial function by sitagliptin therapy is associated with the change in adiponectin. An experimental study also showed that sitagliptin significantly increases circulating levels of adiponectin in OLETF rats [28]. Adiponectin has vasoprotective effects via regulation of endothelial nitric oxide synthase in vascular endothelial cells. Even though our study failed to show a significant increase in adiponectin levels after 1 week of treatment with alogliptin—probably owing to short-term administration—the long-term effect of alogliptin therapy on adiponectin levels needs to be elucidated.

There is mixed evidence for the benefits of improved glycemic control on cardiovascular events and mortality in patients with diabetes mellitus. The 10 years of primary follow-up from the landmark UK Prospective Diabetes Study (UKPDS) [29] and three recent outcome studies (the Action to Control Cardiovascular Risk in Diabetes [ACCORD] [30], Action in Diabetes and Vascular Disease: Preterax and Diamicron Modified Release Controlled Evaluation [ADVANCE] [31], and Veterans Affairs Diabetes Trial [VADT] [32]) all failed to demonstrate that intensive glycemic control reduces cardiovascular events and mortality. In contrast, recent studies showed that a DPP-4 inhibitor may reduce cardiovascular events in patients with diabetes [33]. Experimental studies also showed the favorable action of DPP-4 on vascular cells via a GLP-1-independent mechanism [34]. These data indicate that DPP-4 may have potential for preventing cardiovascular events beyond glycemic control; however, another group reported that vildagliptin has no protective effects on cardiac function in a rat model of post-myocardial infarction heart failure [35]. Thus, the cardiovascular benefits of DPP-4 inhibitors beyond glycemic control should be clarified in a future study.

### Study limitations

There are several important limitations of our study. First, this was an open-label study, and the number of participants enrolled in our study was small. Therefore, a degree of selection bias may have occurred. Second, no widely used method for assessing postprandial hyperlipemia has been established, and so various fat-loading tests, such as oral fat meal, fat cream intake, and intravenous fat load, have been used in previous studies. We used the cookie test, which provided sufficient information about glucose intolerance and postprandial hyperlipemia [13]. Although the cookie provided a fixed amount of fat (28.5 g) per person, the amount of fat given per body surface area was not the same in this study. Therefore, the contribution of each person’s fat metabolism cannot be ruled out as an influential factor. A meal loading test using 30 g fat/m^2^ body surface area showed a greater increase in TG and RLP compared with that using a fixed amount of fat (28.5 g) per person, even in healthy volunteers [7]. The effect of alogliptin on postprandial lipemia after the meal loading test with 30 g fat/m^2^ body surface area would also be informative, especially in patients with diabetes or dyslipidemia.

In conclusion, we demonstrated that inhibition of DPP-4 with alogliptin was effective for reducing postprandial elevation of TG-rich lipoproteins and the accompanying induction of postprandial endothelial dysfunction. Alogliptin may be a useful drug for reducing future cardiovascular disease by ameliorating endothelial dysfunction in the postprandial state, even in low-risk patients.

## Abbreviations

apoB-48: Apolipoprotein B48; AUC: Area under the curve; BMI: Body mass index; DPP-4: Dipeptidyl peptidase IV; FMD: Flow-mediated dilation; GIP: Glucose-dependent insulinotropic polypeptide; GLP-1: Glucagon-like peptide-1; HDL-C: High-density lipoprotein cholesterol; HOMA-IR: Homeostasis model assessment of insulin resistance; LDL-C: Low-density lipoprotein cholesterol; NMD: Nitroglycerin-mediated dilation; RLP-C: Remnant lipoprotein cholesterol; SD: Standard deviation; SE: Standard error; TG: Triglyceride; Total-C: Total cholesterol; VCAM-1: Vascular cell adhesion molecule 1.

## Competing interest

The authors declare that they have no competing interest.

## Authors’ contributions

YN, TM, JO, KN, conceived the study, and participated in its design and coordination and helped to draft the manuscript. YN, TM, YO carried out examinations. NT, KK, HM, KK, HI were involved in drafting the manuscript or revising it critically. All authors read and approved the final manuscript.

## Supplementary Material

Additional file 1 **Table S1.** Postprandial changes in lipid profile, glucose metabolism, and endothelial function in the alogliptin and control groups over time. Click here for file
