# Supplementary material for: Alogliptin ameliorates postprandial lipemia and postprandial endothelial dysfunction in non- diabetic subjects: a preliminary report
Source: Cardiovasc Diabetol. 2013 Jan 9;12:8. doi: 10.1186/1475-2840-12-8 (PMC3557163; doi:10.1186/1475-2840-12-8)
Supplement: Additional file 1 — Table S1. Postprandial changes in lipid profile, glucose metabolism, and endothelial function in the alogliptin and control groups over time. [file 1475-2840-12-8-S1.doc]

**Supplementary Table 1.** Postprandial changes in lipid profile, glucose metabolism, and endothelial function in the alogliptin and control groups over time.

| Variable | Fasting | 2 h | 4 h | 6 h | 8 h |
| --- | --- | --- | --- | --- | --- |
| **Biochemical parameters** |  |  |  |  |  |
| Total-C (mg/dl) |  |  |  |  |  |
| Control | 185 ± 11 | 185 ± 10 | 185 ± 10 | 188 ± 10 | 189 ± 11 |
| Alogliptin | 181 ± 10 | 181 ± 10 | 178 ± 10 | 184 ± 10 | 186 ± 10 |
| LDL-C (mg/dl) |  |  |  |  |  |
| Control | 103 ± 9 | 100 ± 9 | 101 ± 9 | 104 ± 9 | 105 ± 10 |
| Alogliptin | 102 ± 9 | 101 ± 9 | 99 ± 9 | 103 ± 9 | 105 ± 9 |
| TG (mg/dl) |  |  |  |  |  |
| Control | 74 ± 10 | 120 ± 11 | 116 ± 14 | 87 ± 10 | 61 ± 6 |
| Alogliptin | 63 ± 8 | 99 ± 11* | 84 ± 13* | 82 ± 13 | 62 ± 8 |
| RLP-C (mg/dl) |  |  |  |  |  |
| Control | 8.4 ± 1.2 | 12.1 ± 1.2 | 12.4 ± 1.2 | 10.7 ± 0.9 | 8.3 ± 0.7 |
| Alogliptin | 6.7 ± 0.8 | 10.3 ± 1.1* | 9.5 ± 1.4* | 9.1 ± 1.4 | 7.2 ± 0.9 |
| ApoB-48 (μg/ml) |  |  |  |  |  |
| Control | 2.5 ± 0.3 | 5.1 ± 0.5 | 4.2 ± 0.5 | 2.8 ± 0.4 | 1.9 ± 0.2 |
| Alogliptin | 2.2 ± 0.2 | 4.3 ± 0.4* | 3.4 ± 0.4 | 2.5 ± 0.3 | 1.6 ± 0.2 |
| Glucose (mg/dl) |  |  |  |  |  |
| Control | 93 ± 2 | 102 ± 4 | 91 ± 4 | 92 ± 2 | 91 ± 2 |
| Alogliptin | 94 ± 2 | 97 ± 6 | 90 ± 3 | 92 ± 2 | 90 ± 2 |
| Glucagon (pg/ml) |  |  |  |  |  |
| Control | 64 ± 5 | 71 ± 7 | 59 ± 5 | 66 ± 5 | 62 ± 6 |
| Alogliptin | 60 ± 3 | 58 ± 4 | 55 ± 3 | 54 ± 3* | 58 ± 3 |
| GLP-1 (pmol/l) |  |  |  |  |  |
| Control | 3.2 ± 0.2 | 8.4 ± 1.6 | 4.3 ± 0.6 | 3.4 ± 0.5 | 2.7 ± 0.2 |
| Alogliptin | 5.2 ± 0.7* | 12.6 ± 1.0 | 8.5 ± 1.5* | 4.9 ± 0.5* | 4.7 ± 0.9* |
| Soluble VCAM-1(ng/ml) |  |  |  |  |  |
| Control | 628 ± 72 | 622 ± 47 | 588 ± 72 | 637 ± 59 | 609.4 ± 46 |
| Alogliptin | 605 ± 50 | 614 ± 62 | 600 ± 62 | 619 ± 79 | 657 ± 69 |
| Insulin (μIU/ml) |  |  |  |  |  |
| Control | 4.8 ± 0.8 | 21.8 ± 6.4 | 5.2 ± 1.1 | 3.2 ± 0.5 | 2.6 ± 0.3 |
| Alogliptin | 4.9 ± 1.3 | 20.3 ± 5.0 | 5.5 ± 0.9 | 3.0 ± 0.5 | 3.0 ± 0.4 |
| **Endothelial function** |  |  |  |  |  |
| Brachial artery diameter (mm) |  |  |  |  |  |
| Control | 4.02 ± 0.31 | 4.06 ± 0.57 | 4.04 ± 0.30 | 4.0 ± 0.24 | 3.94 ± 0.25 |
| Alogliptin | 3.91 ± 0.35 | 4.00 ± 0.40 | 4.01 ± 0.38 | 3.99 ± 0.39 | 3.97 ± 0.31 |
| %FMD |  |  |  |  |  |
| Control | 11.8 ± 0.6 | 9.4 ± 0.7 | 7.7 ± 0.3 | 10.3 ± 0.5 | 12.3 ± 0.5 |
| Alogliptin | 11.2 ± 0.4 | 9.1 ± 0.3 | 9.2 ± 0.5 | 10.0 ± 0.3 | 11.2 ± 0.5 |
| %NMD |  |  |  |  |  |
| Control | 19.8 ± 5.8 | - | - | - | - |
| Alogliptin | 21.0 ± 6.8 | - | - | - | - |

Data are the mean ± SE. Total-C, total cholesterol; LDL-C, low-density lipoprotein cholesterol; TG, triglyceride; RLP-C, remnant lipoprotein cholesterol; ApoB-48, apolipoprotein B-48; FMD, flow-mediated dilation; NMD, nitroglycerin-mediated dilation; *p < 0.05, vs. control group after treatment for 1 week.
